# Discoidin domain receptor 2 mediates collagen-induced activation of membrane-type 1 matrix metalloproteinase in human fibroblasts

**DOI:** 10.1074/jbc.M116.770057

**Published:** 2017-03-07

**Authors:** Iwona Majkowska, Yasuyuki Shitomi, Noriko Ito, Nathanael S. Gray, Yoshifumi Itoh

**Affiliations:** From the ‡ Kennedy Institute of Rheumatology, Kennedy Institute of Rheumatology, University of Oxford, Roosevelt Drive, Oxford OX3 7FY, United Kingdom and; § Dana-Farber Cancer Institute, Harvard Medical School, Boston, Massachusetts 02215

**Keywords:** arthritis, cancer, collagen, fibroblast, matrix metalloproteinase (MMP), DDR2, MMP-2, MT1-MMP

## Abstract

Membrane-type 1 matrix metalloproteinase (MT1-MMP) is a membrane-bound MMP that is highly expressed in cells with invading capacity, including fibroblasts and invasive cancer cells. However, pathways of MT1-MMP up-regulation are not clearly understood. A potential physiological stimulus for MT1-MMP expression is fibrillar collagen, and it has been shown that it up-regulates both MT1-MMP gene and functions in various cell types. However, the mechanisms of collagen-mediated MT1-MMP activation and its physiological relevance are not known. In this study, we identified discoidin domain receptor 2 (DDR2) as a crucial receptor that mediates this process in human fibroblasts. Knocking down DDR2, but not the β1 integrin subunit, a common subunit for all collagen-binding integrins, inhibited the collagen-induced MT1-MMP-dependent activation of pro-MMP-2 and up-regulation of MT1-MMP at the gene and protein levels. Interestingly, DDR2 knockdown or pharmacological inhibition of DDR2 also inhibited the MT1-MMP-dependent cellular degradation of collagen film, suggesting that cell-surface collagen degradation by MT1-MMP involves DDR2-mediated collagen signaling. This DDR2-mediated mechanism is only present in non-transformed mesenchymal cells as collagen-induced MT1-MMP activation in HT1080 fibrosarcoma cells and MT1-MMP function in MDA-MB231 breast cancer cells were not affected by DDR kinase inhibition. DDR2 activation was found to be noticeably more effective when cells were stimulated by collagen without the non-helical telopeptide region compared with intact collagen fibrils. Furthermore, DDR2-dependent MT1-MMP activation by cartilage was found to be more efficient when the tissue was partially damaged. These data suggest that DDR2 is a microenvironment sensor that regulates fibroblast migration in a collagen-rich environment.

## Introduction

Membrane-type 1 matrix metalloproteinase (MT1-MMP)^2^ is a type I transmembrane proteinase that belongs to the MMP family of enzymes. MT1-MMP is expressed on the cell surface and promotes cellular migration and invasion of different cell types, including various cancer cells, endothelial cells, fibroblasts, mesenchymal stem cells, B-cells, T-cells, monocytes/macrophages, osteoclasts, and epithelial cells, by degrading pericellular extracellular matrix (ECM) to make a migration path (1, 2). MT1-MMP also promotes ECM degradation indirectly by activating other soluble MMPs, including pro-MMP-2 and pro-MMP-13, expanding its proteolytic repertoire (1). Besides ECM, MT1-MMP cleaves other cell-surface molecules such as CD44, ICAM1, syndecan 1, αv integrin chain, tissue transglutaminase, and low-density lipoprotein receptor-related protein 1, modifying cellular functions (1). MT1-MMP was also shown to affect cellular function in a proteolytic activity-independent manner through its cytoplasmic domain by interacting with factor-inhibiting hypoxia-induced factor 1, resulting in increased hypoxia-induced factor 1-mediated transcriptional activity in cancer cells and macrophages (3, 4). Thus, MT1-MMP has a wide variety of means to modify cellular functions, but how MT1-MMP gene is up-regulated in those cells *in vivo* is still not understood.

One of the characteristic activities of MT1-MMP is activation of pro-MMP-2 on the cell surface, and this MT1-MMP-dependent pro-MMP-2 activation was found to be induced by treatment of different cell types with lectins such as concanavalin A (5, 6). It was also found that some inflammatory cytokines, including IL-1 and TNFα, can stimulate this process as well (7), but cytokine-induced pro-MMP-2 activation and MT1-MMP up-regulation were not reproducible in other studies (8, 9). Another cellular stimulus to induce pro-MMP-2 activation is collagen. It was found that monomeric collagen does not stimulate pro-MMP-2 activation, but fibrillar collagen was found to be a stimulus, especially when cells were embedded in a 3D type I collagen lattice (10). As collagen I is a major substrate of MT1-MMP and one of the most abundant matrix components in tissue, this makes collagen a potential physiological inducer of MT1-MMP function. However, the mechanism of collagen-induced MT1-MMP activation has not been clearly elucidated.

Major collagen receptors that can transmit signals to cells are collagen-binding integrins and discoidin domain receptors (DDRs) (11). Collagen-binding integrins include α1β1, α2β1, α10β1, and α11β1 that link ECM molecules to a complex and dynamic network of intracellular adaptors and the cytoskeleton, forming focal contacts and adhesions, which act as a major hub of cell-ECM signaling (11). DDRs are receptor tyrosine kinases (RTKs) in which phosphorylation of their cytoplasmic domain is induced by binding to collagen at their ectodomain (11). DDRs are the only RTKs that recognize solid ECM molecules. There are two members in this subfamily of RTKs, DDR1 and DDR2. In non-transformed tissue, DDR1 is mainly expressed in epithelial cells, whereas DDR2 is found in mesenchymal cells such as fibroblasts. However, the precise roles of DDRs and signaling cascades are still to be investigated.

In this study, we report that DDR2 is the receptor that mediates collagen-induced MT1-MMP expression and its function in human fibroblasts. Interestingly, the role of DDR2 in MT1-MMP activation is rather limited to non-transformed fibroblasts as DDR inhibition does not affect MT1-MMP activation in cancer cells. Our data suggest that DDR2 is a microenvironment sensor that regulates fibroblast migration.

## Results

### Collagen induces MT1-MMP activation in fibroblasts

**Figure 1.**
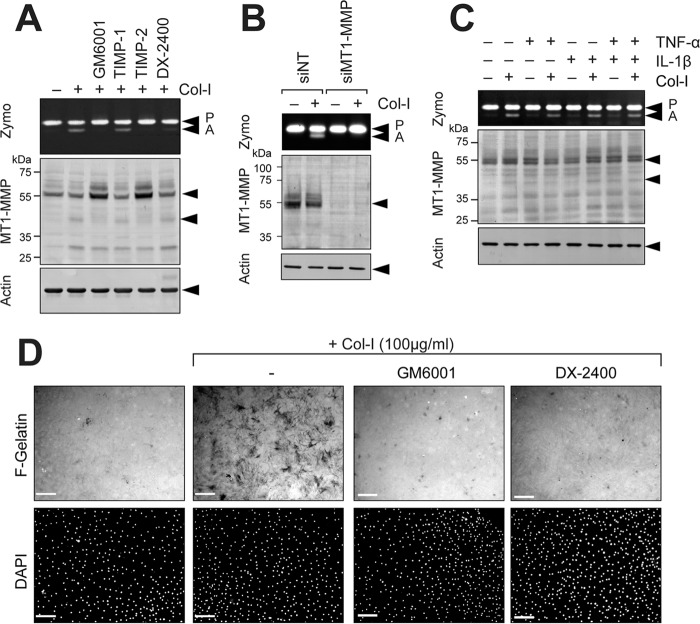
**Collagen activates MT1-MMP functions in RASF.** *A*, human RASF were cultured on a plastic dish and stimulated with or without collagen I (*Col-I*; 100 μg/ml) for 24 h in the presence or absence of GM6001 (10 μm), TIMP-1 (100 nm), TIMP-2 (100 nm), or DX-2400 (500 nm). Media were analyzed by zymography for pro-MMP-2 activation (*Zymo*), and cells were subjected to Western blotting analyses for MT1-MMP and actin. *P*, pro-MMP-2; *A*, active MMP-2. *B*, human RASF transfected with siNT or MT1-MMP siRNA (*siMT1-MMP*) were cultured and stimulated with collagen I (100 μg/ml) for 24 h. Conditioned media and cell lysates were analyzed as in *A. C*, human RASF were stimulated with collagen I, TNF-α (10 ng/ml), and/or IL-1β (10 ng/ml) for 24 h as indicated, and the media and cell lysates were analyzed as in *A. D*, human RASF were subjected to F-gelatin film degradation assay. Cells were cultured on F-gelatin-coated coverslips for 48 h in the presence or absence of collagen I (100 μg/ml) with or without GM6001 (10 μm) or DX-2400 (500 nm). Cells were fixed, nuclei were stained with DAPI, and images were captured with a fluorescence microscope. *Scale bars*, 270 μm.

MT1-MMP is expressed in wide variety of cell types and promotes cellular invasion in tissue. However, pathways of MT1-MMP up-regulation are not clearly understood. It has been shown that fibrillar collagen can induce MT1-MMP function and its expression in various cell types, including fibroblasts (12, 13), endothelial cells (10), and cancer cells (12, 14), and it is possible that collagen is an *in vivo* stimulus of MT1-MMP expression. As shown in Fig. 1*A*, when human rheumatoid synovial fibroblasts (RASF) or human dermal fibroblasts (HDF) were treated with type I collagen, activation of pro-MMP-2 was induced, and GM6001, TIMP-2, and a highly selective MT1-MMP inhibitory antibody, DX-2400, but not TIMP-1, inhibited the activation process. It was noted that, although those cells without collagen stimulation showed detectable levels of pro-MMP-2 and MT1-MMP, pro-MMP-2 activation did not take place. GM6001 and TIMP-2 treatment increased the level of 58-kDa MT1-MMP and reduced the generation of the 44/45-kDa processed form lacking the catalytic domain (15, 16), suggesting that MMP-dependent MT1-MMP processing was induced by collagen and that the increased level of MT1-MMP can only be detected by inhibiting MMPs. It has been shown that functional activation of MT1-MMP is accompanied by generation of the 44/45-kDa processed form either by autocatalytic processing of MT1-MMP itself or by MMP-2 activated by MT1-MMP (15, 16). In addition to those species, a band around 28 kDa was detected upon collagen stimulation. Because it was detected by anti-hemopexin domain antibody, it is likely that this species is a further processed C-terminal fragment of MT1-MMP. Collagen-induced pro-MMP-2 activation was confirmed to be MT1-MMP-dependent as knocking down MT1-MMP completely abolished the process (Fig. 1*B*). Pro-MMP-2 activation was not influenced by inflammatory cytokines, including TNF-α and IL-1, as shown in Fig. 1*C*. One way to detect MT1-MMP activity on the cell surface is gelatin film degradation. As both RASF and HDF express a detectable level of MT1-MMP without any stimulation, it was expected that cells would degrade the gelatin film in an efficient manner. However, they did not degrade the gelatin film very well (Fig. 1*D*). In contrast, collagen stimulation notably induced gelatin film degradation (Fig. 1*D*, +*Col-I*). Gelatin film degradation was also confirmed to be due to MT1-MMP as GM6001 and DX-2400 effectively inhibited gelatin film degradation. These data suggest that MT1-MMP expressed in inert RASF and HDF is not functionally active, and collagen stimulation increases both expression of the enzyme and function of MT1-MMP in fibroblasts.

### DDR2 mediates collagen-induced MT1-MMP activation

**Figure 2.**
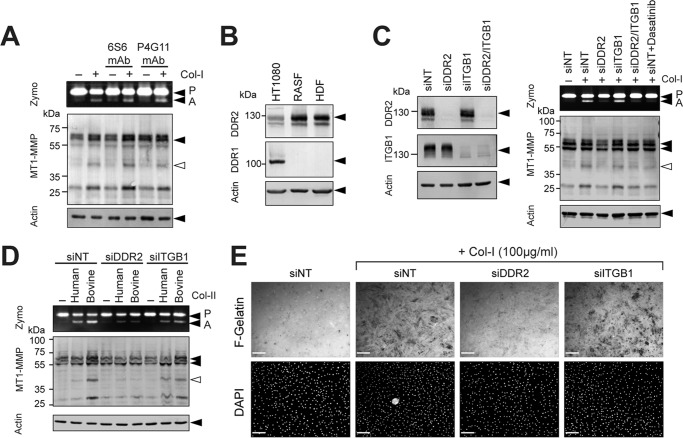
**DDR2 but not integrins mediates collagen signaling to activate MT1-MMP functions in RASF.** *A*, human RASF were stimulated with collagen I (*Col-I*) in the presence or absence of β1 integrin-inhibitory antibody (6S6) or -activating antibody (P4G11) for 24 h in serum-free medium. Conditioned media and cell lysates were analyzed as in *A. B*, cell lysates from HT1080 human fibrosarcoma cells, RASF, and HDF were analyzed for DDR1 and DDR2 expression by Western blotting. RASF and HDF express DDR2 but not DDR1, whereas HT1080 expresses both DDRs. *C*, RASF were transfected with siRNA for DDR2 (*siDDR2*), β1 integrin (*siITGB1*), and/or siNT as indicated. 48 h later, cell lysates were subjected to Western blotting analyses for DDR2, ITGB1, and actin (*left panel*). 48 h after siRNA transfection, cells were also stimulated with collagen I (100 μg/ml) and cultured for a further 72 h. siNT-transfected cells were also treated with dasatinib (*Dasa*; 100 nm). Conditioned media and cell lysates were analyzed as in *A* (*right panel*). *D*, RASF transfected with siNT, siRNA for DDR2, or siRNA for ITGB1 were stimulated with collagen II (*Col-II*) of human origin (100 μg/ml) and bovine origin (100 μg/ml) for 48 h. Conditioned media and cell lysates were analyzed as in *A. E*, RASF transfected with siNT, siRNA for DDR2, or siRNA for ITGB1 were subjected to F-gelatin film degradation assay as in *D. Scale bars*, 270 μm. *P*, pro-MMP-2; *A*, active MMP-2; *Zymo*, zymography.

**Figure 3.**
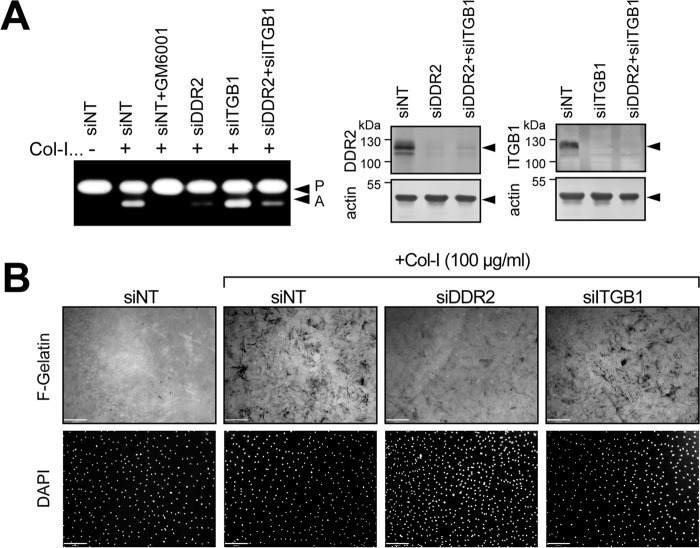
**DDR2 mediates collagen signaling to activate MT1-MMP functions in HDF.** *A*, HDF were transfected with siRNA for DDR2 (*siDDR2*), siRNA for β1 integrin (*siITGB1*), and/or siNT as indicated. 48 h later, cell lysates were subjected to Western blotting analyses for DDR2, ITGB1, and actin (*right panel*).48 h after siRNA transfection, cells were further stimulated with collagen I (*Col-I*) (100 μg/ml) and cultured for a further 48 h. Conditioned media were analyzed by zymography. *B*, HDF transfected with siNT, siRNA for DDR2, or siRNA for ITGB1 were subjected to F-gelatin film degradation assay as in Fig. 2*E. Scale bars*, 270 μm. *P*, pro-MMP-2; *A*, active MMP-2.

Collagen signaling can be mediated by at least two different categories of receptors: one is collagen-binding integrins, and the other is DDRs (11). It has been reported previously that pro-MMP-2 activation is induced by clustering of integrin in ovarian cancer cells (14). We thus examined the effects of function-blocking antibody or -activating antibody for collagen-binding integrins. Because β1 subunit is common to all collagen-binding integrins, we tested anti-β1 integrin antibody clone 6S6 for blocking its function (17) and clone P4G11 for activating integrin function (18). As shown in Fig. 2*A*, neither antibody affected pro-MMP-2 activation in the absence or presence of collagen stimulation. We next examined the potential role of integrins and DDRs by gene silencing. Because RASF and HDF only express DDR2 but not DDR1 (Fig. 2*B*), we knocked down DDR2 and/or β1 integrin. As shown in Fig. 2*C* (*left panel*), transfection of small interfering RNA (siRNA) effectively silenced expression of either DDR2 or β1 integrin by more than 95% in RASF. When those cells were treated with collagen (Fig. 2*C*, *right panel*), cells lacking DDR2 expression were unable to activate pro-MMP-2, whereas knocking down β1 integrin did not have any effects. The tyrosine kinase inhibitor dasatinib is a highly potent DDR inhibitor (19), and it also inhibited induction of pro-MMP-2 activation to a level similar to DDR2 knockdown. Type II collagen is a major component of cartilage matrix, and both human and bovine type II collagen also induced pro-MMP-2 activation in a DDR2-dependent manner (Fig. 2*D*). Collagen-induced gelatin film degradation was also diminished when DDR2 was knocked down but was not diminished by β1 integrin knockdown (Fig. 2*E*, *siDDR2* and *siITGB1*), suggesting that the induction of gelatin film degradation was also a result of collagen signaling through DDR2. We also examined the role of DDR2 in collagen-induced MT1-MMP activation in HDF as shown in Fig. 3. Our data indicate that both collagen-induced pro-MMP-2 activation (Fig. 3*A*) and gelatin film degradation (Fig. 3*B*) were DDR2-dependent.

**Figure 4.**
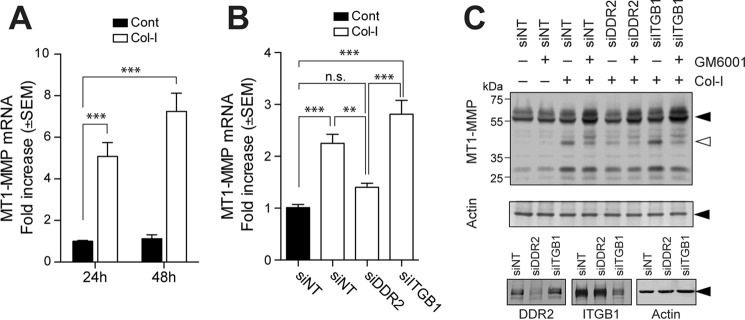
**Role of DDR2 in collagen-induced MT1-MMP gene expression.** *A*, human RASF were stimulated with or without collagen I (*Col-I*) (*open bars* or *closed bars*, respectively) for 24 or 48 h, and the level of MT1-MMP gene expression was examined by qPCR (*n* = 6). ***, *p* > 0.001. *B*, RASF transfected with siNT, siRNA for DDR2 (*siDDR2*), or siRNA for β1 integrin (*siITGB1*) were stimulated with collagen (*open bars*). *Error bars* represent S.E. (*n* = 6). ***, *p* > 0.001; **, *p* > 0.01; *n.s.*, not significant. *C*, RASF transfected with siNT, siRNA for DDR2, or siRNA for ITGB1 were stimulated with collagen I (100 μg/ml) in the presence or absence of GM6001for 72 h. Cell lysates were subjected to Western blotting analyses for MT1-MMP, actin, DDR2, and ITGB1. *Cont*, control.

Collagen-induced MT1-MMP activation was accompanied by increased MT1-MMP gene expression. Collagen stimulation significantly increased MT1-MMP mRNA as shown in Fig. 4*A*. Interestingly, the mRNA level induced by collagen was sustained for a long time as there was a slightly greater increase at 48 compared with 24 h. This collagen-induced gene expression was confirmed to be mediated by DDR2 as DDR2 knockdown, but not β1 integrin knockdown, significantly reduced the mRNA level (Fig. 4*B*). The increased mRNA does not apparently correlate with the protein level of the 58-kDa band but did correlate with the appearance of the 44/45-kDa processed form (Fig. 2). Our observation supports the previous notion that the appearance of this processed form is correlated with increased MT1-MMP function (15, 16). When cells were stimulated with collagen in the presence of GM6001, both MT1-MMP and MMP-2 were inhibited, resulting in a decrease of the 44/45-kDa band and an increase in bands of the intact form of MT1-MMP around 58 kDa (Fig. 4*C*). DDR2 knockdown, but not β1 integrin knockdown, notably decreased the generation of the 44/45-kDa form and increased the full-length 58-kDa band (Fig. 4*C*).

### DDR2 signaling is necessary for cell-surface collagen degradation by MT1-MMP

**Figure 5.**
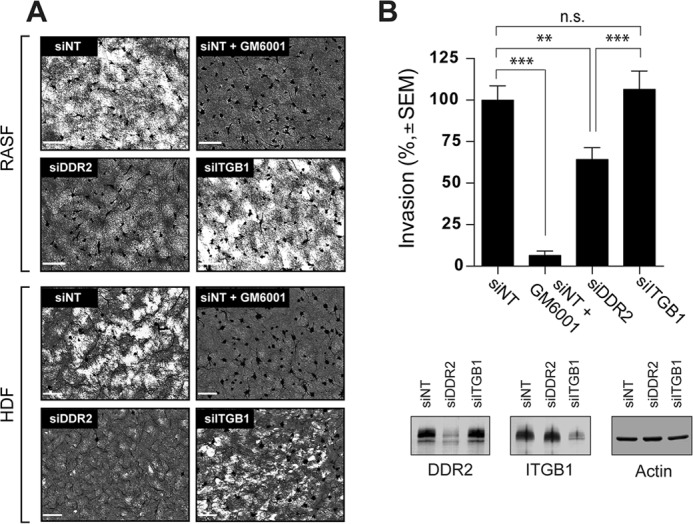
**Role of DDR2 in MT1-MMP-dependent collagen film degradation and collagen invasion.** *A*, human RASF or HDF transfected with siNT, siRNA for DDR2 (*siDDR2*), or siRNA for β1 integrin (*siITGB1*) were subjected to collagen film degradation assay as described under “Experimental Procedures” (*left panel*). Conditioned media from this assay were analyzed by zymography. *P*, pro-MMP-2; *A*, active MMP-2. *Scale bars*, 270 μm. *B*, human RASF transfected with siNT, siRNA for DDR2, or siRNA for ITGB1 were subjected to Transwell collagen invasion assay as described under “Experimental Procedures” (*upper panel*). Levels of DDR2, ITGB1, and actin in transfected cells were analyzed by Western blotting (*bottom panel*). These are combined data of three independent experiments (*n* = 6 for each experiments). *Error bars* represent S.E. ***, *p* > 0.001; *n.s.*, not significant.

When RASF are cultured on collagen film, they degrade the film in an MT1-MMP-dependent manner (8, 9). Collagen is a substrate for MT1-MMP, but it is also a stimulatory ligand for DDR2 to activate MT1-MMP. We thus next examined whether collagen signaling through DDR2 is involved in cellular degradation of collagen. As shown in Fig. 5*A*, RASF or HDF degraded collagen film in an efficient manner (non-targeting siRNA (*siNT*)), and it was inhibited by GM6001 (*siNT* + *GM6001*). Knocking down DDR2 (*siDDR2*), but not β1 integrin (*siITGB1*), efficiently inhibited collagen film degradation.

We then examined effect of knockdown of DDR2 and β1 integrin on collagen invasion by RASF. As shown in Fig. 5*B*, DDR2 knockdown significantly inhibited cellular invasion, whereas β1 integrin knockdown showed no effect.

**Figure 6.**
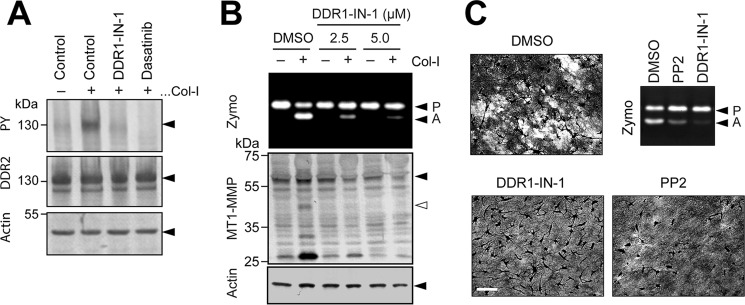
**Effect of pharmacological inhibition of DDR kinase on MT1-MMP functions.** *A*, HEK293 cells were transfected with DDR2 expression plasmid and stimulated with collagen I (*Col-I*) (100 μg/ml) in the presence or absence of DDR1-IN-1 (5 μm) or dasatinib (100 nm) for 4 h. Cell lysates were analyzed by Western blotting for phosphotyrosine (*PY*) (4G10), DDR2, and actin. *B*, human RASF were stimulated with collagen I (100 μg/ml) in the presence or absence of DDR1-IN-1 at 2.5 or 5.0 μm for 48 h. Conditioned media and cell lysates were analyzed as in Fig. 2*A. C*, RASF were subjected to collagen film degradation assay in the presence or absence of DDR1-IN-1 (5 μm) or the Src tyrosine kinase inhibitor PP2 (10 μm). Culture media from the assay were analyzed by zymography (*Zymo*). *P*, pro-MMP-2; *A*, active MMP-2.

It has been reported that DDR1 can act as an adaptor molecule for MT1-MMP to localize the enzyme on a linear invadosome at collagen substrate (20), and this may provide a potential mechanism of DDR-dependent cellular invasiveness. We thus next examined whether the inhibitory effect of DDR2 knockdown on collagen film degradation is due to a lack of DDR2 molecule or a lack of collagen signaling through DDR2 by using a highly selective DDR kinase inhibitor, DDR1-IN-1. DDR1-IN-1 inhibits DDR1 at an IC_50_ of 105 nm and DDR2 at an IC_50_ of 413 nm, and none of the other tyrosine kinases are inhibited (21). Because RASF express DDR2 but not DDR1 (Fig. 1), DDR1-IN-1 inhibits only DDR2 tyrosine kinase. As shown in Fig. 6*A*, DDR1-IN-1 inhibited collagen-induced phosphorylation as effectively as dasatinib. Addition of DDR1-IN-1 inhibited pro-MMP-2 activation in a dose-dependent manner (Fig. 6*B*). DDR1-IN-1 also inhibited collagen film degradation by RASF (Fig. 6*C*, *DDR1-IN-1*). It has been shown that Src is activated downstream of DDR2 activation, and a Src inhibitor, PP2, also similarly inhibited collagen film degradation (Fig. 6*C*, *PP2*) as well as collagen-induced pro-MMP-2 activation (Fig. 6*C*). We therefore concluded that it is the DDR2 signaling that results in activation of MT1-MMP-dependent cell-surface collagen degradation.

### Proteolytic modification of collagenous tissue may be prerequisite for efficient DDR2-mediated activation of MT1-MMP

**Figure 7.**
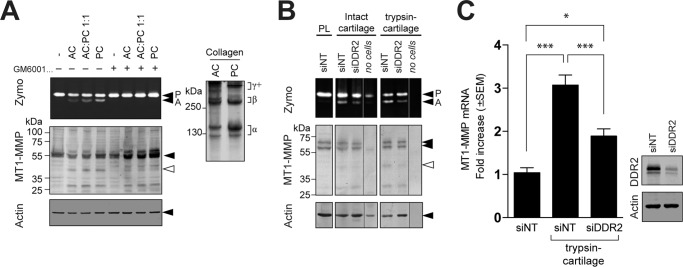
**Effect of partial damage on collagen and cartilage for activation of MT1-MMP function.** *A*, human RASF were treated with AC and/or PC (total of 100 μg/ml each) in the presence or absence of GM6001 (10 μm) as indicated. Conditioned media and cell lysates were analyzed as in Fig. 2*A*. AC and PC used in this experiment were analyzed by SDS-PAGE under reducing conditions (50 μg/lane). The collagen bands were visualized by staining with Coomassie Brilliant Blue (*right panel*). *B*, disks of bovine nasal cartilage were treated with or without trypsin (100 μg/ml) for 18 h. Cartilage was washed and treated with soybean trypsin inhibitor (200 μg/ml) for 24 h to inhibit trypsin activity followed by extensive washing with PBS. RASF transfected with siNT or siRNA for DDR2 (*siDDR2*) were cultured on the cartilage disks for 96 h. Conditioned media and cell/cartilage disk extracts were analyzed as in Fig. 2*A*. The gel/blot lanes from cartilage alone without cells (*no cells*) were spliced and placed next to the samples of the cells transfected with siRNA for DDR2. Note that the no-cells sample from intact cartilage has pro-MMP-2 most likely derived from chondrocytes in the tissue. *C*, human RASF transfected with siNT or siRNA for DDR2 were cultured on cartilage treated with trypsin and analyzed for MT1-MMP gene expression by qPCR (*left panel*). Levels of DDR2 in the cells were analyzed by Western blotting (*right panel*). *Error bars* represent S.E. (*n* = 6). ***, *p* > 0.001; *, *p* > 0.1. *Zymo*, zymography, *P*, pro-MMP-2; *A*, active MMP-2.

DDR2-binding sites in collagens II and III have been identified by screening a triple-helical peptide library (22). Interestingly, based on the collagen fibrillar structure solved by X-ray fiber diffraction analysis (23), none of those DDR-binding sites are exposed on the surface of collagen fibrils, suggesting that intact collagen fibrils cannot stimulate DDR2 efficiently. However, one of the binding sites can be exposed upon cleavage of telopeptides. To test this hypothesis, RASF were cultured on a film of intact acid-extracted collagen (AC), pepsin-treated collagen lacking telopeptide (PC), or a 1:1 mixture of AC and PC. As shown in Fig. 7*A* (*right panel*), AC contains a higher proportion of highly cross-linked chains (γ+), whereas the major forms of PC are monomeric (α) and dimeric (β) because the telopeptide region is where cross-links occur. AC did not induce MT1-MMP-dependent pro-MMP-2 activation in an efficient manner, whereas PC effectively induced pro-MMP-2 activation in a proportion-dependent manner, suggesting that proteolytic action on a telopeptide lesion of a collagen molecule in cartilage or other tissues may trigger efficient induction of DDR2-dependent collagen signaling in pathophysiological conditions.

In cartilage, the gaps between collagen fibrils are largely filled by aggrecan proteoglycan, and it is possible that aggrecan limits accessibility of DDR2 to collagen. To examine this, RASF were cultured on cartilage that had been treated with or without trypsin. We and others previously reported that this treatment effectively removes aggrecans without degrading collagen (24, 25), although trypsin is also likely to remove other minor components in cartilage. As shown in Fig. 7*B*, when RASF were cultured on control cartilage, a moderate level of pro-MMP-2 was activated in the medium, and DDR2 knockdown effectively decreased activation. In contrast, when RASF were cultured on trypsin-treated cartilage, a noticeably higher proportion of pro-MMP-2 was activated compared with control cartilage, and DDR2 knockdown again effectively decreased activation. The increased pro-MMP-2 activation was accompanied by increased MT1-MMP mRNA (Fig. 7*C*), and DDR2 knockdown inhibited this significantly. Taking these data together, we concluded that cartilage collagen in intact tissue may not trigger DDR2 activation in an effective manner and that it requires partial damage of the matrix to become an efficient stimulator for DDR2 to up-regulate MT1-MMP expression and functions in RASF.

### Collagen-induced MT1-MMP activation in cancer cells is not mediated by DDRs

**Figure 8.**
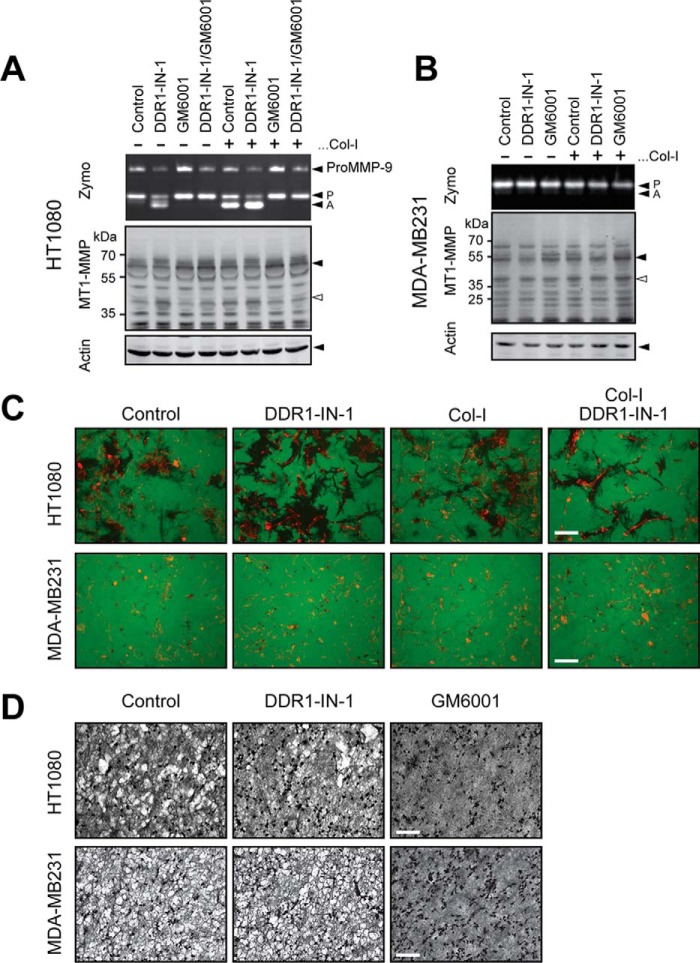
**Effect of pharmacological inhibition of DDRs in cancer cells on collagen-induced MT1-MMP activation.** *A*, HT1080 cells were treated with collagen I (*Col-I*) in the presence or absence of DDR1-IN-1 (5 μm) and/or GM6001 (10 μm) as indicated. Conditioned media and cell lysates were analyzed as in Fig. 2*A. B*, MDA-MB231 cells were treated with collagen in the presence or absence of DDR1-IN-1 (5 μm) and/or GM6001 (10 μm) as indicated. Exogenous pro-MMP-2-containing conditioned medium from HEK293 cells stably expressing pro-MMP-2 was added in the medium as MDA-MB231 does not produce pro-MMP-2. Conditioned media and cell lysates were analyzed as in *A. C*, HT1080 or MDA-MB231 cells were subjected to gelatin film degradation assay in the presence or absence of DDR1-IN-1 (5 μm) and/or collagen I (100 μg/ml) as indicated. *Scale bars*, 270 μm. *D*, HT1080 or MDA-MB231 cells were subjected to collagen film degradation assay in the presence or absence of DDR1-IN-1 (5 μm) or collagen I (100 μg/ml) as indicated. *Scale bar*, 270 μm. *Zymo*, zymography, *P*, pro-MMP-2; *A*, active MMP-2.

Collagen-dependent MT1-MMP activation can also be seen in cancer cells, and cancer cells degrade collagen matrix using MT1-MMP. We thus examined whether DDR-dependent collagen signaling also mediates MT1-MMP activation in cancer cells. We examined HT1080 human fibrosarcoma cells and MDA-MB231 human breast cancer cells. HT1080 cells express both DDR1 and DDR2 (Fig. 2*B*), and MDA-MB231 cells are also known to express both DDRs (20, 26–28). As shown in Fig. 8*A*, collagen stimulation generates the active form of MMP-2 in HT1080 cells. However, treatment with DDR1-IN-1 alone induced processing of pro-MMP-2, and co-treatment of cells with collagen and DDR1-IN-1 additively enhanced pro-MMP-2 activation. In MDA-MB231 cells, neither DDR1-IN-1 nor collagen stimulation induced pro-MMP-2 activation (Fig. 8*B*). We next examined the effect of DDR1-IN-1 on gelatin film degradation activity by those cells (Fig. 8*C*). In HT1080 cells, DDR1-IN-1 treatment clearly increased the area of gelatin degradation, whereas collagen treatment did not have much effect. Cells co-treated with collagen and DDR1-IN-1 showed more defined degradation in comparison with control cells or collagen alone-treated cells but not as much as the cells treated with DDR1-IN-1 alone. MDA-MB231 cell-degraded gelatin exhibited a dotlike pattern (typical invadopodia), and DDR1-IN-1 did not show any effect. Upon collagen stimulation with or without DDR1-IN-1, the pattern of degradation was not significantly changed. We finally examined the effect of collagen film degradation by these cells (Fig. 8*D*). In HT1080 cells, DDR1-IN-1 treatment showed a slight inhibitory effect, whereas GM6001 completely inhibited degradation. In MDA-MB231 cells, DDR1-IN-1 did not affect collagen degradation at all, whereas GM6001 inhibited it completely. Cumulatively, these results suggest that the role of collagen and DDRs in MT1-MMP regulation in cancer cells is different from that in fibroblasts, and the role of DDR2 in MT1-MMP activation seems to be limited to stromal fibroblasts.

## Discussion

MT1-MMP is a crucial invasion promoter for fibroblasts such as RASF (8, 9, 29) and HDF (30). However, the mechanisms of up-regulation of MT1-MMP gene and functions *in vivo* are not clearly understood. Here we report that collagen-induced MT1-MMP activation is mediated through DDR2, and this may be an *in vivo* mechanism of MT1-MMP up-regulation in human fibroblasts.

It has been reported that collagen-induced MT1-MMP activation is a result of induction of integrin clustering (16). However, in our experiments, knocking down β1 integrin, a common subunit of all collagen-binding integrins, did not have an impact on collagen-induced MT1-MMP activation, whereas DDR2 knockdown efficiently inhibited the process. DDR2-dependent activation of MT1-MMP gene/functions is a result of activation of DDR2 signaling because the selective DDR kinase inhibitor DDR1-IN-1 inhibited the activation. It has been reported that Src-dependent phosphorylation of DDR2 at tyrosine 740 is important for DDR2 signaling upon collagen stimulation (31). Our data showed that PP2, an inhibitor of Src, also effectively inhibited MT1-MMP activation, further supporting this idea.

Collagen-induced MT1-MMP activation has been observed in many different cell types, including cancer cells (12, 14). Therefore, we also examined whether DDR2 plays a role in HT1080 human fibrosarcoma cells and MDA-MB231 human breast cancer cells. Although human fibroblasts express DDR2 but not DDR1 (Fig. 2), HT1080 human fibrosarcoma cells express both DDR1 and DDR2 (Fig. 2*B*) (20, 26, 27). DDR1-IN-1, which inhibits both kinases, failed to inhibit MT1-MMP activation in these cancer cells. In HT1080 cells, DDR1-IN-1 treatment even promoted MMP-2 activation and gelatin film degradation by up-regulating the MT1-MMP level. These data suggest that a role of DDR2-mediated collagen signaling in this aspect is limited to non-transformed stromal fibroblasts. Collagen-induced MT1-MMP gene expression was also shown in non-transformed epithelial cells (32) that lack DDR2 expression. Therefore, there are other mechanisms by which collagen up-regulates MT1-MMP in different cell types. Nevertheless, a major pathway of collagen-induced MT1-MMP activation in human fibroblasts is through DDR2.

Our data indicate that DDR2 signaling is also required for fibroblasts to degrade collagen. DDR2 knockdown or treating cells with PP2 or DDR1-IN-1 effectively inhibited collagen film degradation by RASF. Because RASF express detectable MT1-MMP even without collagen stimulation, it was unexpected that inhibition of DDR2 signaling showed such a major effect on collagen degradation. One possible explanation is that the basal level of MT1-MMP is too low to express collagenolytic activity. However, the increase of MT1-MMP levels upon collagen stimulation is only up to 2-fold. Thus, this may not explain the induction of collagenolytic activity upon recognition of collagen through DDR2. Previously it was reported that collagen-induced functional activation of MT1-MMP in MCF7 cells expressing MT1-MMP is a consequence of inhibition of clathrin-dependent endocytosis due to interaction of collagen and the hemopexin domain of MT1-MMP (33). However, this scenario is also unlikely in fibroblasts because MT1-MMP/collagen interaction would not be inhibited by DDR1-IN-1 or PP2. Another possibility includes induction of localization of MT1-MMP to the collagen fibril. It is possible that cells may need to recognize collagen substrate by DDR2 to present MT1-MMP to the collagen fibrils by directing secretory vesicles containing MT1-MMP (34). If so, collagen degradation on the cell surface is not due to simple enzyme/substrate interaction but involves a step in which the cells recognize the substrate by DDR2. It is possible that such relocalization may be accompanied by functional activation of the enzyme as well. Nevertheless, further detailed mechanisms are to be investigated in the future.

In the early stages of arthritis, aggrecan removal precedes collagen degradation in cartilage. Previously we have reported that RASF can invade aggrecan-depleted cartilage better than the intact cartilage (8). It has also been reported that aggrecan removal makes cartilage more susceptible to MMP-1-dependent collagenolysis *in vitro* (24). Furthermore, knock-in mice with a mutation in the interglobular domain of aggrecan that are resistant to cleavage by aggrecanase are protected from cartilage erosion in surgically induced osteoarthritis (35). Thus, aggrecan likely prevents access of collagenolytic proteinases to collagen fibril in the tissue, and aggrecan loss may be a trigger for cartilage erosion during the development of arthritis. Our data in this report indicate that trypsin treatment, which effectively removes aggrecan and other minor cartilage components (25), enhanced the ability of cartilage to stimulate MT1-MMP activation. The components removed by trypsin presumably limit DDR2 activation by restricting interaction of DDR2 with collagen in the tissue. The detailed molecular arrangements among DDR2, cartilage collagen, aggrecan, and other components are to be investigated in the future.

The DDR1- and DDR2-binding sites in type II and III collagens were identified previously (22, 36). The crystal structure of the discoidin domain of DDR2 bound to triple-helical collagen peptide has also been solved (37). However, according to the quaternary structure of fibrillar collagen from rat tail tendon solved by Orgel *et al.* (23), those DDR-binding sites identified in collagen monomer are not exposed on the surface of collagen fibrils. Therefore, most fibrillar collagens in healthy intact tissue may not be able to activate DDRs effectively. Failed *in vitro* collagen fibrillogenesis in the presence of soluble ectodomain of DDR1 or DDR2 supports this notion (38). Interestingly, one of the DDR2-binding sites in type II collagen (amino acids 775–801) can be exposed upon removal of non-helical telopeptides, which mask this region, and indeed we found that pepsin-treated collagen, which had lost the telopeptide region, stimulated MT1-MMP activation more readily than the intact collagens (Fig. 7*A*). It has been shown that proteolytic cleavage of telopeptides of type I collagen greatly influences their fibril assembly and the rigidity of collagen gel (39). Neutrophil elastase, cathepsin G, lysosomal cysteine proteinases, and MMP-3 are known to cleave telopeptide of cartilage collagen and are up-regulated in the inflammatory conditions such as in RA (40). Although activation of other RTKs is regulated by both gene expression and bioavailability of soluble ligands, it is possible that activation of DDRs relies on modification of existing inert collagen molecules by proteolytic enzymes as the ligand collagens are abundantly present in tissue.

In RA joints, MMP-13 is highly up-regulated in RASF at the pannus-cartilage junction (41). MMP-13 was also shown to be a downstream gene of DDR2 signaling (42). Because it has been reported that both MT1-MMP and MMP-13 are co-up-regulated at pannus-cartilage junctions (41), it is possible that this is due to DDR2-dependent cartilage collagen signaling in RASF during RA development. The co-expression of MT1-MMP and MMP-13 may accelerate cartilage erosion as MT1-MMP activates pro-MMP-13 on the cell surface (41, 43). Thus, DDR2 activation at the site may play a crucial role in joint destruction, and DDR2 is potentially a novel therapeutic target for RA. Previously administration of the tyrosine kinase inhibitor imatinib was shown to prevent arthritis development in mice (44–46). Imatinib was originally developed as an inhibitor for the oncogene BCR-ABL tyrosine kinase, and it is an approved drug for chronic myelogenous leukemia (47). However, imatinib was also shown to inhibit DDR1 and DDR2 as well (19). Although imatinib is not a selective inhibitor for DDRs, it is possible that inhibition of DDR2 by imatinib contributes to the suppressive effect in arthritis development, and DDR2-selective inhibition may be a potential novel therapeutic strategy for RA therapy in the future.

In the present study, we identified DDR2 as the receptor that mediates collagen-induced MT1-MMP gene expression and functional activation in human fibroblasts. This seems to be a unique mechanism for stromal fibroblasts because in cancer cells and epithelial cells collagen-induced MT1-MMP activation is mediated by other mechanisms. Further investigation of the DDR2-independent mechanisms will be an important task to understand microenvironment-driven cellular invasion *in vivo*.

## Experimental procedures

### Reagents

Anti-MT1-MMP (222-1D8) antibody was kindly provided by Prof. Motoharu Seiki (University of Tokyo, Tokyo, Japan), DX-2400 was a gift from Dyax Corp. (Burlington, MA). Anti-MT1-MMP antibody (EP1264Y) was from Abcam (Cambridge, UK), anti-DDR2 antibody (AF2538) was from R&D Systems (Abingdon, UK), anti-actin antibody (C-19) was from Santa Cruz Biotechnology (Santa Cruz, CA), anti-β1 integrin antibodies (AB1952, 6S6, and P4G11) were from Millipore (Watford, UK). Anti-phosphotyrosine (4G10) and anti-mouse and anti-goat alkaline phosphatase-conjugated antibodies were from Sigma-Aldrich, and anti-rabbit alkaline phosphatase-conjugated antibody was from Promega (Southampton, UK). SMARTpool ON-TARGETplus siRNAs for MT1-MMP, DDR2, integrin β1 subunit (ITGB1), and siNT were from Thermo Scientific (Northumberland, UK). Recombinant TNF-α and IL-1β were from Peprotech (London, UK), PureCol collagen type I was from Advanced BioMatrix (Leimuiden, The Netherlands), CellMatrix type I-A collagen was from Nitta-Gelatin Inc. (Osaka, Japan), human and bovine type II collagens were from Chondrex Inc. (Redmond, WA), dasatinib was from LC Laboratories (Woburn, MA), trypsin was from Sigma-Aldrich, and DDR1-IN-1 was synthesized as reported previously (21).

### Cell culture and siRNA transfection

RASF were isolated from synovium tissue from RA patients as described previously (8). HDF were isolated from skin of a normal donor and cultured in the same way as RASF. HT1080 and MDA-MB231 cells were from ATCC. All of these cells were maintained in Dulbecco's modified Eagle's medium with 4.5 g/liter glucose supplemented with 10% fetal bovine serum and antibiotics (Lonza Biologics, Slough, UK). RASF or HDF were transfected with 5 nm siRNAs using INTERFERin (VWR International Ltd., Lutterworth, UK) according to the manufacturer's protocol.

### Western blotting and zymography

Cell lysates were prepared in SDS-PAGE sample buffer (with 2-mercaptoethanol) and subjected to Western blotting as described previously (48). Serum-free conditioned media were subjected to gelatin zymography (48). Densitometry analysis was performed using Phoretix 1D software.

### Gelatin film degradation assay

Glass coverslips were coated with 50 μg/ml Alexa Fluor 488-conjugated gelatin (F-gelatin) as described previously (48, 49). Cells (3 × 10^4^) were cultured atop of the coverslips for 48 h in serum-free medium with or without PureCol collagen added in the medium at 100 μg/ml. Cells were then fixed in 3% paraformaldehyde in PBS for 15 min and stained with DAPI (Sigma-Aldrich). Images were taken with a Nikon Eclipse TE2000-E inverted fluorescence microscope equipped with a charge-coupled device camera.

### Collagen film degradation assay

Collagen film degradation was performed as described previously (48, 49). Briefly, 12-well plates were coated with 100 μl of 2 mg/ml chilled and neutralized PureCol collagen and incubated at 37 °C for 1 h to induce fibril formation. RASF (5 × 10^4^) were cultured on the collagen film in serum-free medium for 72 h. For some samples, GM6001 (Elastin Products Co., Owensville, MO) was added to culture medium at 10 μm as indicated. Cells were removed by trypsin with EDTA, and the collagen layer fixed with 3% paraformaldehyde in PBS for 15 min followed by staining with Coomassie Brilliant Blue R-250. Images were taken with a Nikon microscope with a 4× objective lens.

### Transwell invasion assay

The Transwell invasion assay was performed as described previously (50) using a 12-well insert with 8-μm-pore membrane Transwells (VWR International Ltd.). The Transwell was coated with 50 μl of CellMatrix and PureCol collagens mixture (1:2, 2 mg/ml) and incubated at 37 °C to set the collagen. RASF (5 × 10^4^) were seeded in the upper chamber and further cultured for 72 h. Invaded cells were stained with DAPI, imaged with fluorescence microscopy, and analyzed by Volocity software (PerkinElmer Life Sciences).

### Quantitative PCR (qPCR)

Total RNA was isolated with a Micro RNeasy RNA extraction kit (Qiagen, Crawley, UK) and was reverse transcribed to cDNA with a High-Capacity cDNA Reverse Transcription kit (Life Technologies). MT1-MMP mRNA levels were measured by real time qPCR using MT1-MMP TaqMan probe (Hs00237119-m1) and Ribosomal RNA Control Reagents (Life Technologies). Relative changes in MT1-MMP mRNA were calculated using the comparative cycle threshold method.

### Cartilage preparation

Frozen and thawed bovine nasal septum cartilage was cut to ∼7 × 5 × 3-mm pieces and incubated with or without 100 μg/ml trypsin for 18 h at 37 °C. Cartilage explants were treated with 200 μg/ml soybean trypsin inhibitor (Sigma-Aldrich) for 18 h to completely inactivate trypsin and then extensively washed in DMEM with 10% FBS. RA synovial fibroblasts were attached to cartilage explants and overlaid with serum-free DMEM.

## Author contributions

I. M. performed the experiments shown in Figs. 1–4 and 7 and wrote the manuscript. Y. S. performed the experiments shown in Figs. 5 and 6 and edited the manuscript. N. I. performed the experiments for Fig. 8. N. S. G. provided DDR1-IN-1 and edited the manuscript. Y. I. conceived and oversaw the project and wrote the manuscript. All authors reviewed the results and approved the final version of the manuscript.

## Footnotes

2 The abbreviations used are:

MT1-MMP: membrane-type 1 matrix metalloproteinase
MMP: matrix metalloproteinase
ECM: extracellular matrix
DDR: discoidin domain receptor
RTK: receptor tyrosine kinase
RASF: rheumatoid arthritis synovial fibroblasts
HDF: human dermal fibroblasts
siNT: non-targeting siRNA
AC: acid-extracted collagen
PC: pepsin-treated collagen lacking telopeptide
RA: rheumatoid arthritis
ITGB1: integrin β1 subunit
F-gelatin: Alexa Fluor 488-conjugated gelatin.
